# Phenotypic characterization of a female patient with retinitis pigmentosa caused by a homozygous X-linked *RPGR*^*ORF15*^ mutation

**DOI:** 10.1016/j.ajoc.2025.102290

**Published:** 2025-02-23

**Authors:** Marlene Saßmannshausen, Elisa A. Mahler, Sandrine H. Künzel, Constanze L. Kochs, Frank G. Holz, David Rosenkranz, Hanno J. Bolz, Philipp Herrmann

**Affiliations:** a Department of Ophthalmology, University Hospital Bonn, Germany; b MVZ of the University Medical Center Mainz GmbH, Germany; c Bioscientia Human Genetics, Institute for Medical Diagnostics GmbH, Ingelheim, Germany

**Keywords:** *RPGR*, Retinitis pigmentosa, RP3, Gene-augmentation-therapy, Homozygosity-*RPGR*^*ORF15*^, Phenotype-genotype-correlation, X-inactivation

## Abstract

**Purpose:**

To describe a detailed phenotypic expression of a homozygous female with retinitis pigmentosa (RP) within a consanguineous family revealing an extremely rare genetic constellation with possible implications for future emerging therapies in addressing inherited retinal dystrophies.

**Observations:**

Multimodal retinal imaging including wide field fundus photography, fundus autofluoresence (FAF), high-resolution spectral domain optical coherence tomography (SD-OCT) imaging, functional testing comprising visual fields and electroretinogram as well as genetic testing were performed in two consanguine cases of RP.

A 44-year-old female patient was referred for evaluation and counseling for potential treatment options presenting with night blindness and visual field defects since early childhood. Extended ophthalmologic examination including multimodal retinal imaging and functional testing showed a clinical presentation of a RP phenotype. The accompanying 50-year-old paternal uncle reported similar visual symptoms and was diagnosed with RP during adolescence. In multimodal retinal imaging, both patients presented a similar phenotype and comparable disease severity with a global photoreceptor loss and decreased FAF signal. In the uncle, there was evidence for central residuals of the photoreceptor band on SD-OCT imaging and a patchy FAF pattern. Genetic testing revealed a rare constellation of a homozygous RP GTPase Regulator protein (*RPGR*^*ORF15*^) mutation in the female patient.

**Conclusions and importance:**

This detailed phenotype-genotype correlation presents a novel clinical presentation of a rare homozygous *RPGR*^*ORF15*^ mutation in a female patient that exhibits severe retinal degeneration similar to affected males and therefore, considering they don't have a wildtype allele, may be suitable for inclusion in upcoming therapeutic treatment trials.

## 1 Introduction

Inherited retinal diseases (IRD) are a phenotypically and genetically heterogeneous group of monogenic diseases, which can lead to blindness and visual impairment at an early age. The most common IRD subgroup is retinitis pigmentosa (RP) with an estimated prevalence of up to 1:3000 that is inherited as either autosomal-dominant (30–40 %), autosomal-recessive (50–60 %) or also X-linked (5–15 %).^1^^,^^2^ Up to 80 % of the X-linked retinitis pigmentosa (XLRP) cases are linked to mutations detected on the *RPGR* gene.^3^, ^4^, ^5^, ^6^, ^7^ *RPGR* encodes for the RP GTPase Regulator protein (*RPGR*), which is responsible for the ciliary transport of metabolites between inner and outer photoreceptor segments.^8^ Mutations in exons 1–14 account for only 25 % of all disease causing mutations, while the large exon open reading frame 15 (*ORF15*) harbors up to 80 % of disease-causing *RPGR* mutations.^3^ Due to its repetitive purine-rich sequence, genetic testing has been challenging, but different strategies can be applied to detect mutations in *ORF15*.^9^

Novel therapeutic approaches like gene augmentation therapy are aiming to slow disease progression, or even to revert the pathological phenotype to some extent. In this context, official clinical approval of Luxturna (voretigene neparvovec) for a single gene causing RP (*RPE65*) by the Federal Food and Drug Administration in 2017 has given new hopes also for other IRDs. The currently four registered phase III clinical trials are further demonstrating the growing interest in developing treatment for *RPGR*-related RP (clinicaltrials.gov.: NCT04671433, NCT04794101, NCT03584165, NCT04850118).^3^

The phenotypical presentation of *RPGR*-related IRD is broad and comprises a classical rod-cone RP phenotype with early onset of night blindness and difficulties with dark adaptation resulting from mutations most commonly found in exons 1–14.^5^^,^^10^ However, mutations in *RPGR* can also lead to cone-rod dystrophy reported in around 20 % of patients with day-time vision problems, photophobia, dyschromatopisa and blurred central vision.^5^^,^^8^^,^^10^^,^^11^

Representing an X-linked disease, most patients are hemizygous males while the clinical presentation of female carriers varies considerably.^6^^,^^7^^,^^12^ Females carriers present a spectrum of retinal disease severity, ranging from mild or subclinical retinal phenotype to severe retinal degeneration and significant visual impairment.^7^

Herein, we demonstrate a comprehensive multimodal retinal imaging-based clinical phenotyping of a female patient from a consanguineous Turkish family with a homozygous pathogenic *RPGR* variant.

## 2 Case report

**Fig. 1:**
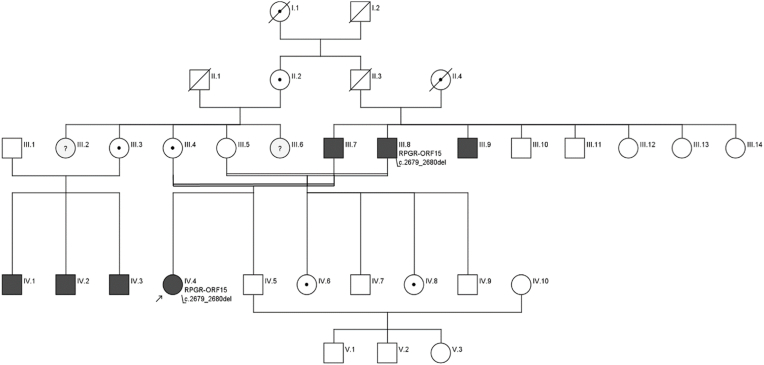
Family pedigree.

Detailed patients’ characteristics including the identified mutation as well as the corresponding family pedigree are provided in Table 1 and Fig. 1.

**Table 1:** Patients’ characteristics.

ID	Age	Gender	Refraction [dpt] (sph/cyl) OD/OS	BCVA OD/OS	Gene	Zygosity	Nucleotide	Class	Protein	Reference
III.8	50	m	+1.00/-0.75	FC	*RPGR*^*ORF15*^	hemi.	c.2679_2680del	5	p. (Glu894Glyfs*184)	[^14^^,^^18^]
			+1.50/-1.75	20/200						
IV.4	44	f	+0.75/-0.75	20/200	*RPGR*^ORF15^	homo.	c.2679_2680del	5	p. (Glu894Glyfs*184)	[^14^^,^^18^]
			+0.75/-0.75	20/200						

Abbreviations: f: female, m: male, OD: right eye, OS: left eye, BCVA: best-corrected visual acuity, FC: finger-counting, homo.: homozygous, hemi.: hemizygous.

**Fig. 2:**
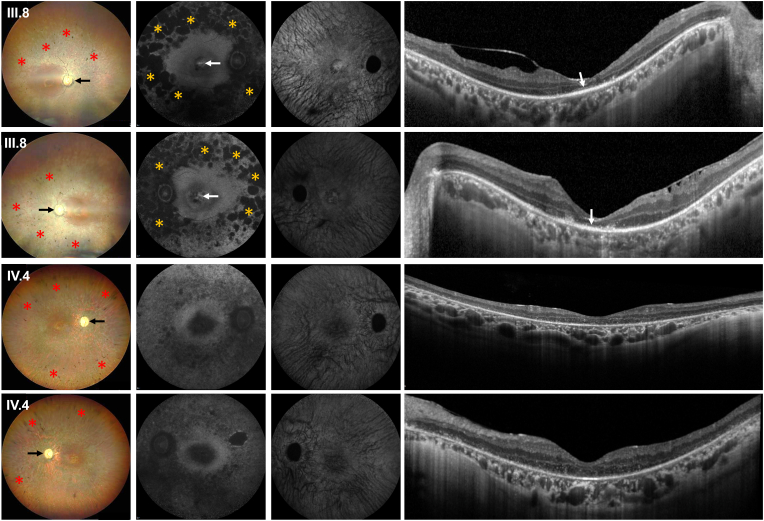
**Clinical presentation of patients** Fig. 2: Clinical phenotype of the right and left eye of the 50-year-old uncle (III.8) with the hemizygous *RPGR*^*ORF15*^ and the 44-year-old female patient (IV.4) with the homozygous *RPGR*^*ORF15*^ mutation. In both patients multimodal retinal imaging reveals typical RP characteristics including retinal pigmentary changes in form of bone-spicule and pigment clumpings in the midperiphery (red asterisks) and a waxy pallor of the optic disc (black arrows) in wide-field color fundus photography (first column). The 55° blue-fundus-autofluorescence (FAF, second column) imaging is globally reduced. In patient III.8 there is a paracentral ring of increased FAF with a patchy-like reduced FAF in the mid-periphery of the retina (yellow asterisks) and a well-delineable hyperautofluorescent ring at the foveal center (white arrows). The 55° near-infrared autofluorescence imaging (third column) appears overall similarly reduced in both patients, while horizontal spectral-domain optical coherence tomography (SD-OCT) imaging (last column) reveals a global loss of the photoreceptor layers with evidence of single residuals of photoreceptor cells at the foveal center in patient III.8 (white arrows). (For interpretation of the references to color in this figure legend, the reader is referred to the Web version of this article.)

A 50-year-old male person (III.8, pedigree) reported night blindness since childhood at the age of 10 and the diagnosis of RP was made at the age of 25, when he noticed a progressive visual impairment with loss of contrast vision and increasing reading problems. At his initial presentation in our IRD clinic, vision was counting fingers and 20/200, residual visual fields were smaller than 5° and photopic and scotopic responses on full-field electroretinogram (ERG) were extinguished. Clinical examination revealed a clinical RP phenotype with retinal pigmentary changes, a paracentral ring of increased FAF with a patchy-like reduced FAF in the mid-periphery of the retina and a well-delineable hyperautofluorescent ring at the foveal center. Spectral domain optical coherence tomography (SD-OCT) imaging demonstrated single centrally remaining photoreceptors. This patient is the paternal uncle of a homozygous female who was referred to our clinic after her initial presentation.

The 44-year-old female patient (IV.4, pedigree) was referred for an extended ophthalmological workup to our IRD service reporting about night blindness and visual field defects since early childhood. At the age of 11 years, she had been diagnosed with RP in Turkey. She further reported progressive visual field loss over the last 10 years. No syndromic findings were reported. Several of her relatives (mostly living in Turkey) have also been diagnosed with RP in the past. Her parents were first-degree cousins. With best-corrected visual acuity (BCVA) of 20/200 in both eyes, clinical examination revealed typical RP characteristics including bilateral posterior subcapsular cataract, retinal pigmentary changes, decreased FAF in the mid-periphery of the retina as well as a global loss of the photoreceptor bands in SD-OCT imaging.^7^ Her functional testing also revealed outer visual field borders of less than 5° in visual field testing and extinguished photopic and scotopic responses on ERG testing.

Genetic testing was performed by target enrichment with IDT xGen® Exome Research Panel V2.0 (IDT Integrated Technologies, Coraville, IA, USA) using additional *RPGR*^ORF15^-specfic xGen Lockdown Probes (IDT Integrated Technologies, Coraville, IA, USA), sequencing on Illumina NextSeq500 system (Illumina, San Diego, C, USA), quality-based data filtering incl. sequence read trimming (in house software qlip V1.2) and data evaluation with the SeqNext module software (V5.3.4 JSI Medical Systems, Ettenheim, Germany).^13^ Performed genetic testing was non-CLIA (Clinical Laboratory Improvements Amendments) certified. Molecular testing revealed a hemizygous status in the male patient for a previously described pathogenic frameshift variant (c.2679_2680del, p. Glu894Glyfs∗184, class 5, RefSeq Number: NM_001034853.2) in *ORF15* of the *RPGR* gene resulting in a stop codon after the inclusion of 183 unrelated residues.^14^ Our female patient presented the same variant homozygously.

## 3 Discussion

In this report we present a detailed multimodal retinal imaging-based characterization of a very rare homozygous *RPGR*^ORF15^ variant detected in a female RP patient with a clinically similar RP disease severity compared to the patient's paternal uncle revealing a hemizygous variant within a consanguine family. The presented homozygous female case highlights the effect of the total absence of any functional protein being produced due to the homozygous gene mutation and therefore needs to be regarded in contrast to heterozygous *RPGR* female carrier presenting with a remaining wildtype allele and varying clinical extent of X-chromosome inactivation.^12^^,^^15^

In X-linked RP, female carriers may typically present with a heterogenous clinical phenotype ranging from being unaffected, mildly affected without functional significance or being severely affected. Apart from the effect of different variants, this wide range of clinical presentation can be explained by the varying extent of X-chromosome inactivation.^15^ In our female patient, with a homozygous mutation in *RPGR*, X-inactivation does not play a role as both alleles are equally affected and no functional protein is being expressed. This also explains the severe phenotype of our female patient that is similar to male patients with a hemizygous mutation in *RPGR*.

While an X-linked homozygous *RPGR*^ORF15^ variant has previously been described in an Iranian consanguineous family with RP,^16^ our report for the first time demonstrates a very detailed multimodal retinal imaging phenotyping as well as genotypic correlation in a female homozygous patient. Especially with regards to upcoming genetic therapies in *RPGR* patients, homozygous *RPGR* female carriers might potentially benefit from gene augmentation therapy treatments as well. However, additional studies are needed here to prove this claim in further detail. To address this, detailed phenotype to genotype correlations are crucial, not just to characterize the disease, but also to counsel patients also with regards to their own families and children and finally also to identify potential candidates for upcoming gene therapy based therapeutic approaches with special regards to current ongoing trials on *RPGR* gene augmentation therapy. For female carriers, consideration of the ratio of X-chromosome inactivation and their correlation to clinical disease severity is currently under discussion.^12^^,^^15^^,^^17^ This ratio can help to predict long-term disease severity but might also help to identify future candidates for an early disease intervention, who potentially profit most effectively from future therapies.

To conclude, this case study emphasizes the need of a refined phenotype to genotyping correlation in IRD affected patients to enable an individualized counseling of patients and families as well as to identify potential candidates for future upcoming therapeutic approaches at an early stage of disease.

## CRediT authorship contribution statement

**Marlene Saßmannshausen:** Writing – review & editing, Writing – original draft, Visualization, Validation, Supervision, Resources, Project administration, Methodology, Investigation, Formal analysis, Data curation, Conceptualization. **Elisa A. Mahler:** Writing – review & editing, Data curation. **Sandrine H. Künzel:** Writing – original draft. **Constanze L. Kochs:** Investigation. **Frank G. Holz:** Writing – review & editing, Supervision, Funding acquisition, Conceptualization. **David Rosenkranz:** Writing – review & editing, Investigation. **Hanno J. Bolz:** Writing – review & editing, Methodology, Investigation. **Philipp Herrmann:** Writing – review & editing, Writing – original draft, Visualization, Validation, Supervision, Resources, Project administration, Methodology, Investigation, Funding acquisition, Formal analysis, Data curation, Conceptualization.

## Patient consent

Written consent has been obtained from all patients.

## Authorship

All authors attest that they meet the current ICMJE criteria for authorship.

## Funding

N/A.
